# Nodal metastases in papillary thyroid microcarcinoma: prevalence and risk factors in 311 patients

**DOI:** 10.1007/s00428-025-04312-1

**Published:** 2025-11-18

**Authors:** Maria Letizia Lai, Priscilla Baldussu, Jacopo Caschili, Luigi Minerba, Maria Luisa Altana, Giovanni Pinna, Pietro Giorgio Calò, Clara Gerosa, Daniela Fanni

**Affiliations:** 1 Department of Medical Sciences and Public Health, Division of Pathology (AOU Di Cagliari), University of Cagliari, Cagliari, Italy; 2 Department of Medical Sciences and Public Health (AOU Di Cagliari), University of Cagliari, Cagliari, Italy; 3 Nuova Casa Di Cura, Cagliari, Decimomannu Italy; 4 Department of Surgical Sciences (AOU Di Cagliari), University of Cagliari, Cagliari, Italy

**Keywords:** Papillary thyroid microcarcinoma (PTMC), Extrathyroidal extension (ETE), Lymph node metastasis

## Abstract

Papillary thyroid microcarcinoma (PTMC) is generally considered low risk due to its favourable prognosis; however, in some cases, it presents aggressive features such as lymph node metastasis and extrathyroidal extension (ETE). The aim of this study was to investigate the pathological factors that influence prognosis in PTMC with the purpose of refining risk stratification based on our cohort of 311 cases. We performed a retrospective analysis based on anonymous data from 311 PTMC samples (tumours ≤ 1.0 cm in size) collected between 2016 and 2024. We examined several variables, including gender, histological subtype, tumour size, ETE, lymph node metastasis, and thyroiditis. The evaluations followed the eighth edition of the American Joint Committee on Cancer (AJCC) staging system. We used Pearson’s chi-square test for univariate analysis and binary logistic regression for multivariate analysis. A *p*-value less than 0.05 was considered statistically significant. In our cohort, 45 patients (14.5%) had lymph node metastases. Male sex (OR = 3.3132; 95% CI = 1.5554–7.0574; *p* = 0.002), age < 45 years (OR = 2.4974; 95% CI = 1.2228–5.1006; *p* = 0.012), multicentricity (OR = 2.9351; 95% CI = 1.4314–6.0182; *p* = 0.003) and vascular invasion (OR = 3.5184; 95% CI = 1.3044–9.4905; *p* = 0.013) are found to be independent risk factors for lymph node metastases. The tall cell histological subtype (OR = 3.897; 95% CI = 1.6649–9.122; *p* = 0.002) emerged as an independent predictor of ETE. Although PTMC is commonly considered an indolent neoplasm, some cases may present aggressive features that require careful prognostic evaluation. The identification of independent risk factors may improve clinical decision-making and therapeutic strategies for patients with PTMC.

## Introduction

Defined only by a size of 10 mm or less according to the World Health Organization (WHO), papillary thyroid microcarcinoma (PTMC) should not be considered a distinct subtype, but rather a prognostic descriptive report [1]. PTMC accounts for approximately 70–90% of well-differentiated thyroid tumours, often (36%) detected during autopsy or thyroidectomy performed for other reasons, such as benign thyroid diseases, especially when the macroscopic examination of the thyroid sample is accurate [2]. PTMC has a predominantly female distribution, with an age range of 27–75 years, and the most affected anatomical area is the middle third of the right or left thyroid lobe [3]. From a histological appearance, the characteristic neoplastic cells, which show nuclear clearing, overlapping, grooves, and pseudoinclusions, are found within or at the periphery of a scar-like infiltrative pattern. Sometimes a thick fibrous capsule with focal calcifications is detectable. The architecture may be totally follicular, partially papillary, or meet the criteria for specific histological subtypes [4–9].

Commonly associated with an excellent prognosis, PTMC has usually been considered clinically insignificant, to the point that a group of experts has even proposed renaming it papillary microtumour of the thyroid (PMiT) for those patients with incidental PTMC with a negligible risk of developing adverse clinical events [10]. However, despite the excellent prognosis, PTMC still presents lymph node and distant metastases even at the time of diagnosis [5, 10–13] and, in rare cases, lethal outcomes [14, 15]. In fact, lymph node metastasis is one of the most worrying factors associated with local recurrence, distant metastasis [16, 17] and fatal outcome [18] in PTC. According 2025 ATA guidelines, small lymph-node metastases in ≤ 5 lymph node is considered a low-risk condition and lymph node dissection is recommend in case of clinically involved lymph nodes [19]. Nevertheless, the therapeutic approach for lymph node metastases remains controversial in PTMC [20], with some professionals suggesting aggressive excision [21, 22], while others deny any impact of small subclinical lymph node metastases on patient survival [23].

Therefore, a scientific debate has been initiated on the clinical-pathological characteristics that can effectively influence the risk of adverse outcomes [8, 24–30]. Aggressive clinical behaviour in PTMC has been widely associated with specific histological subtypes of papillary thyroid carcinoma (PTC), vascular invasion, location within the thyroid gland (peripheral vs. central), molecular profile, and family history of PTC [8, 28, 29, 31–36].

PTCs larger than 5–7 mm have been shown to have a high risk of recurrence, lymph node metastasis, and extrathyroidal extension (ETE), while multicentric disease, peripheral (subcapsular) location, and ETE [29] have been associated with a higher risk of recurrence and lymph node metastasis at diagnosis. Other authors have stated the existence of a rule for specific histopathological variables, such as tumour subtyping of primary or metastatic deposits in lymph nodes [5]. Hence, as there is still a need to better predict the behaviour of PTMC, we present our series of 311 PTMCs, focusing on identifying the pathological features that influence the risk of poor prognosis.

## Materials and methods

### Inclusion criteria

We performed a spontaneous retrospective study based on the review of anonymously collected cluster data. From 2016 to 2024, 311 thyroid specimens came to the Unit of Pathology of the University Hospital of Cagliari for histopathological analysis. All these tumors were ≤ 1.0 cm in size and thus received the final pathological diagnosis of PTMC. We carried out quantitative and descriptive analyses that included gender, average age, histological subtype, tumour size, ETE, lymph node metastases, and presence of thyroiditis.

### Histopathologic features

The dimension of the carcinoma was based on gross pathology description and plain microscopic measure of the tumor on the slides at the microscope. Tumours carrying two or more foci of carcinoma in the thyroid were defined as multicentric, in which cases, the tumor size was defined as the larger diameter of the primary tumor according to the eighth edition of the American Joint Committee on Cancer (AJCC) [37]. The presence of vascular and tumor capsular invasion was observed when intravascular tumour cells adhered to the vessel wall, or when the tumor capsule was involved. The number of lymph nodes, the dimensions and the number of lymph node metastases were based on reviewing the gross and microscopic examination of the surgical specimen by the pathologist. Tumour cells invading beyond the thyroid capsule into extrathyroidal tissues were recorded as ETE. Thyroiditis was reported when confirmed at pathological examination.

### Statistical analysis

Collected clustered data express nominal variables in percentage and frequency. Age was expressed as mean ± standard deviation. To highlight differences between groups of specific variables, Jamovi software v 2.3.28 was used. Univariate analyses were performed using Pearson’s chi-square test for nominal variables. Multivariate analyses were performed using binary logistic regression to assess the independent associations of the factors that were statistically significant in the univariate analyses. Results are expressed as odds ratio (95% CI) and *p*-value. A *p*-value < 0.05 was considered statistically significant.

## Results

Of the 311 patients, 246 (79%) were women and 65 men (21%), with a minimum age of 15 years and a maximum age of 77 years (mean age 49.8 years ± 14.3).

**Fig. 1:**
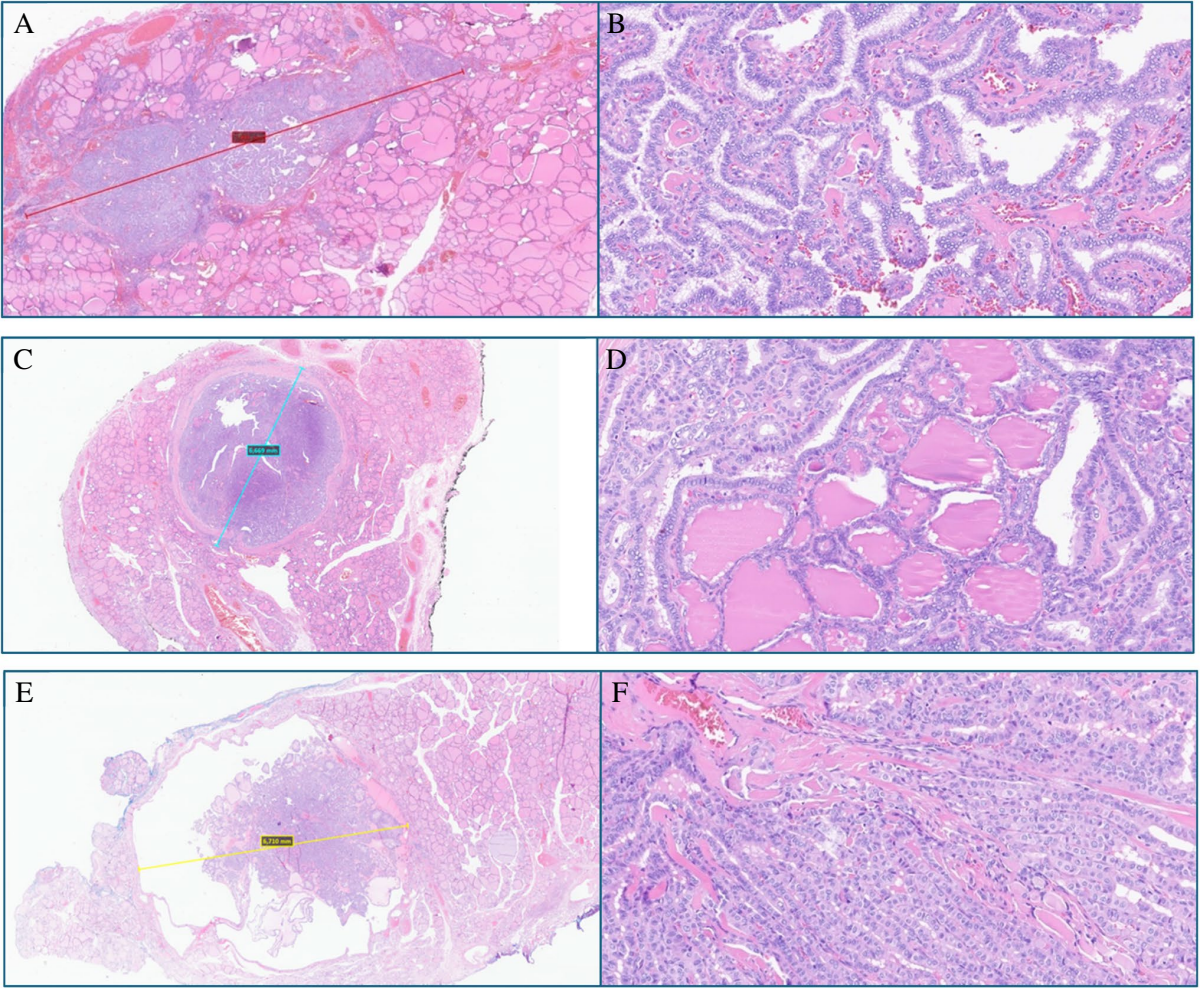
Low- and high-power of three examples of PTMCs. **A**, **B** A classic subtype measuring 7 mm; H&E. **C**, **D** A 6669 mm in size of follicular subtype; H&E. **E**, **F** Tall cell features within a nodule of 6710 mm; H&E

The lesions stood unicentric in 216 cases (69.5%) and multicentric in 95 cases (30.5%); of all cases, the histological subtypes found were 189 classic (60.77%), 63 follicular (20.26%), and 59 tall cells (18.97%) (Fig. 1).

ETE was found in 68 cases (21.9%), and vascular invasion was present in 27 cases (8.7%); lymph node metastases were found in 45 cases (14.5%), while thyroiditis was identified in 161 cases (51.8%).

Univariate analyses revealed a statistically significant association with lymph node metastasis of PTMC and the following variables: gender, age < 45 years, multicentric nodules, ETE, vascular invasion, and nodule size < 5 mm (Table 1).

**Table 1:** Univariate analysis of lymph-node metastasis

	Lymph-node metastasis No	Lymph-node metastasis Yes	*p*-value
	**(*N* = 266)**	**(*N* = 45)**	
Gender (M)	48/266 (18%)	17/45 (37.8%)	*p* = 0.003
Age (< 45)	87/266 (32.7%)	23/45 (51.1%)	*p* = *0.017*
Multicentric nodules	72/266 (27.1%)	23/45 (51.1%)	*p* = *0.001*
Classic	163/266 (61.3%)	26/45 (57.8%)	*p* = 0.656
Follicular	55/266 (20.7%)	8/45 (17.7%)	*p* = 0.655
Tall cell	218/266 (81.9%)	34/45 (75.5%)	*p* = 0.311
ETE	51/266 (19.2%)	17/45 (37.8%)	*p* = 0.005
VI	17/266 (6.4%)	10/45 (22.2%)	*p* < 0.001
Thyroiditis	142/266 (53.4%)	19/45 (42.2%)	*p* = 0.166
Nodule size < 5 mm	109/266 (41%)	11/45 (24.4%)	*p* = 0.035

*M* male, *ETE* extrathyroidal extension, *VI* vascular invasion

From the multivariate analysis, it occurred that male sex (OR = 3.3132; 95% CI = 1.5554–7.0574; *p* = 0.002), age < 45 years (OR = 2.4974; 95% CI = 1.2228–5.1006; *p* = 0.012), multicentricity (OR = 2.9351; 95% CI = 1.4314–6.0182; *p* = 0.003), and the presence of vascular invasion (OR = 3.5184; 95% CI = 1.3044–9.4905; *p* = 0.013) are found to be independent risk factors for lymph node metastases (Table 2).

**Table 2:** Multivariate logistic regression analysis of lymph-node metastasis

						95% Confidence interval	
Predictor	Esteem	SE	*Z*	*p*-value	Odds ratio	Lower	Upper
Gender (M)	1.198	0.386	3.105	0.002	3.3132	1.5554	7.0574
Age (< 45)	0.915	0.364	2.512	0.012	2.4974	1.2228	5.1006
Multicentric nodules	1.077	0.366	2.939	0.003	2.9351	1.4314	6.0182
ETE	0.528	0.395	1.334	0.182	1.6948	0.7807	3.6789
VI	1.258	0.506	2.485	0.013	3.5184	1.3044	9.4905
Nodule size < 5 mm	-0.149	0.432	-0.345	0.730	0.8617	0.3695	2.0095

*M* male, *ETE* extrathyroidal extension, *VI* vascular invasion

Although the association between histological subtype and lymph node metastasis was not statistically significant (Table 1), a statistically significant association emerged between the presence of ETE and the classic (*p* = 0.019) and tall cell (*p* < 0.001) histological subtypes (Table 3); subsequent multivariate analysis demonstrated that only the tall cell histological subtype (OR = 3.897; 95% CI = 1.6649–9.122; *p* = 0.002) was an independent risk factor for ETE (Table 4).

**Table 3:** Univariate analysis of ETE

	ETE Yes	ETE No	*p*-value
	(*N* = 68)	(*N* = 243)	
Classic	33/68 (48.5%)	156/243 (64.2%)	*p* = 0.019
Follicular	10/68 (14.7%)	53/243 (21.8%)	*p* = *0.198*
Tall cell	43/68 (63.2%)	209/243 (86%)	*p* < *0.001*

**Table 4:** Multivariate logistic regression analysis of ETE

						95% Confidence interval	
Predictor	Esteem	SE	Z	*p*-value	Odds ratio	Lower	Upper
Classic	0.114	0.394	0.290	0.772	1.121	0.5175	2.429
Tall cell	1.360	0.434	3.135	0.002	3.897	1.6649	9.122

## Discussion

In 2003, a group of authoritative experts recommended renaming PTMC as PMiT in the absence of aggressive characteristics [10]. Since the risk of recurrence is approximately 1–2%, the American Thyroid Association (ATA) guidelines classify PTCM as low risk [19]. According to the most recent series, PTMC presents with lymph node metastases in approximately 5% of cases, exceptionally with distant metastases and only rarely with fatal outcomes [8, 14, 15, 28]. In this scenario, where there is a continuing need to predict a better prognosis for PTMC, we focused on the pathological characteristics that influence the risk of unfavourable behaviour in our series of 311 PTMCs.

In scientific literature, lymph node metastasis and ETE have already been considered aggressive features and the most important predictors of recurrence [38]. In our series of 311 PTMCs, lymph node metastasis was found in 45 cases (14.5%) and ETE in 68 cases (21.9%). Univariate analyses disclosed a statistically significant correlation between lymph node metastasis and factors such as sex, multicentric disease, ETE, vascular invasion, and tumour size in our series. In line with previous research [38–42], our multivariate analysis identified several independent risk factors for lymph node metastasis, including male sex, age < 45 years, multicentricity and vascular invasion. While some authors [38] have claimed that the presence of thyroiditis is a protective factor and others have associated it with aggressive PTC behaviour [43], our series did not show such a connection. Furthermore, in our series, the association between ETE and the tall cell and classic histological subtypes appeared statistically significant, although histological subtype and lymph node metastasis appeared unrelated. Further multivariate analyses revealed that only the tall cell histological subtype was an independent risk factor for ETE.

Aliyev et al. [12] further refined the definition of PMiT based on exclusion criteria, as they appear to show negligible malignant potential. Therefore, to meet the diagnosis of PMiT, patients must be over 19 years of age, the overall size of multiple tumour foci must be less than 10 mm, the tumour must be found incidentally during investigations performed for other reasons, while aggressive histological features, such as vascular invasion, tall cells, and hobnail cells, the already mentioned ETE and, if known, a positive family history of thyroid cancer must be considered exclusion criteria. In our cases, 304 patients (97.7%) were over 19 years of age, all tumours met the criterion of incidental discovery, while vascular invasion was present in 27 cases (8.68%), tall cell histotype in 59 cases (18.97%) and none had a family history of thyroid cancer. We counted 95 (30.54%) multicentric tumours. Although the definition of PMiT [10, 12, 44, 45] recommended adding the average of the respective tumour diameters in cases of multiple tumours, the eighth edition of the AJCC [37] still recommends measuring the largest nodule in the staging system; therefore, in our series, we strictly followed the method prescribed by the AJCC.

In 2015, Zhang et al. [46] advised considering risk factors for prophylactic central lymph node dissection in male patients with tumor size exceeding 5 mm or carrying and extracapsular spread. Although whether the resection is required for PTMC is controversial, the prognosis is less favorable in patients with lymph node metastasis or extrathyroidal invasion. Due to the high risk for lymphatic spread in PTMC associated with ETE, regional lymph node resection with lobectomy was recommended [47]. In our series, we accounted 120 (33.1%) tumors measuring less than 5 mm, and among them, 16 displayed aggressive features, 11 (9.2%) lymph node metastases, 2 (1.6%) vascular invasion, and 5 (4.2%) ETE.

Analysis of our data has highlighted several critical aspects that contribute to a better understanding of the biological behaviour of PTMC and its clinical implications. Firstly, the 14.5% rate of lymph node metastasis observed in our cohort is in line with recent series [8, 28] challenges the notion that PTMC is always an indolent neoplasm. Although historically classified as a low-risk condition, the presence of lymph node metastasis in a considerable percentage of cases highlights the need for careful prognostic assessment and a more personalized therapeutic approach. Both univariate and multivariate analyses conducted within our cohort confirmed that several factors, including male sex, age under 45 years, multicentric disease, and vascular invasion, are independent risk factors for lymph node metastases [19]. Another noteworthy finding concerns the tall cell subtype, which emerged as an independent predictor of ETE. This reinforces the idea that PTMC with tall cell characteristics may behave more aggressively, requiring closer clinical monitoring and potentially influencing surgical decisions [7, 29]. Furthermore, our study confirms that aggressive features may also be present in tumours smaller than 5 mm, challenging the assumption that microcarcinomas below this threshold always follow an indolent course. The detection of lymph node metastases, vascular invasion, and ETE in a subgroup of these small tumours suggests that size alone should not be the sole determining factor in therapeutic strategies [29]. Finally, our findings indicate that there is no significant correlation between thyroiditis and tumour aggressiveness, contradicting some previous studies that suggested a potential protective effect. This discrepancy highlights the need for further investigation into the role of thyroiditis in the progression of PTMC, as well as its impact on risk stratification and patient management [43].

These findings underline the importance of refining current PTMC risk stratification models and considering a more personalized approach to treatment, particularly for patients with high-risk pathological features. Our results suggest that implementing such criteria, together with adherence to AJCC guidelines [37], may improve diagnostic accuracy and management strategies. It is important to emphasise that aggressive features such as lymph node metastasis and ETE, even in small tumours less than 5 mm in size, should be considered when determining the need for more proactive surgical interventions, such as prophylactic lymph node dissection.

In conclusion, while most cases of PTMC appear to follow an indolent course, our study highlights a subgroup of cases that present a higher risk of recurrence and metastasis. Future research should focus on refining risk stratification models and personalizing therapeutic approaches to effectively balance the benefits of intervention with the risk of overtreatment.
